# On the Uses of Alpha Naphthol

**Published:** 1897-12

**Authors:** 


### Selections.

### ON THE USES OF ALPHA NAPHTHOL.

DR. J. v. MAXIMOWITSCH (*Deutsche Arch. für klin. Med.*, lvii., 3-4,) points out the advantages of alpha naphthol over betanaphthol. It is three times less toxic and a three times stronger antiseptic.

Dr. v. Maximowitsch gives the following as useful formulæ :

1. To disinfect the intestine, a solution in castor oil is best, because evacuation of intestine and antisepsis go together.

| | | |
|---|---|---|
| ℞ | Alpha Naphthol | gr. xlv. |
| | Chloroform | mv. |
| | Ol. Menth. Pip. | mii. |
| | Ol. Ricini, ad | ℥iii. |

One tablespoonful for a dose.

2. For choleraic diarrhea and true cholera.

| | | |
|---|---|---|
| ℞ | Alpha Naphthol | ʒiss. |
| | Chloroform | ʒss. |
| | Ol. Menth. Pip. | miv. |
| | Ol. Ricini, ad | ℥iii. |

One to four tablespoonfuls daily.

Another method of employing the drug, in some ways preferable, owing to the sharp burning taste of alpha naphthol, is to give it in compressed tabloids.

Small tabloids :

| | |
|---|---|
| Alpha Naphthol | gr. iv. |
| Pulv. Rhei. | gr. i. |
| Ext. Belladonna | gr. $\frac{1}{6}$. |
| Tinct. Cinnamon. | mii. |

For one tabloid. One to two tabloids for a dose ; about ten tabloids to be taken a day.

Large tabloids :

| | |
|---|---|
| Alpha Naphthol | gr. viii. |
| Pulv. Rhei. | gr. ii. |
| Ext. Belladonna | gr. $\frac{1}{6}$. |
| Tinct. Cinnamon. | mii. |

For one tabloid. One to two for a dose ; six to ten tabloids to be taken a day.

In enteric fever Dr. v. Maximowitsch uses tabloids freshly prepared, according to the following formulæ :

| | |
|---|---|
| Alpha Naphthol | gr. iv. |
| Phenacetin | gr. iii. |
| Pulv. Rhei. | gr. i. |
| Tr. Cinnamon | mii. |

For one tabloid. One or two tabloids six times a day.

In severe cases of enteric he uses one of the following powders :

| | |
|---|---|
| Alpha Naphthol | gr. viii. |
| Bismuth Salicylate. | gr. ii. |
| Pulv. Rhei | gr. ii. |
| Ext. Bellad | gr. $\frac{1}{6}$. |
| Pulv. Cinnamon | gr. iii. |

In cachet. One four to six times daily.

| | |
|---|---|
| Or, Alpha Naphthol | gr. viii. |
| Phenacetin | gr. vi. |
| Quin. Brom. | gr. ii. |
| Pulv. Rhei | gr. i. |

In cachet. One six times daily. —*Treatment.*

